# Impact of the Eduball method on cognitive creativity, motor creativity, and motor fitness during physical education classes in 8- to 9-year-old children

**DOI:** 10.3389/fpubh.2025.1660650

**Published:** 2025-09-23

**Authors:** Maryna Khorkova, Łukasz Bojkowski, Agata Korcz, Jana Krzysztoszek, Marlena Łopatka, Dagny Adamczak, Michał Bronikowski

**Affiliations:** ^1^Department of Physical Activity Teaching, Poznan University of Physical Education, Poznan, Poland; ^2^Department of Psychology, Poznan University of Physical Education, Poznan, Poland

**Keywords:** Eduball method, cognitive creativity, motor creativity, motor fitness, early school-aged children

## Abstract

**Introduction:**

Creativity is increasingly recognized as a crucial skill across various fields. Although schools are placing more emphasis on fostering creativity, physical education (PE) often remains overlooked. The Eduball method, which combines physical activity with cognitive challenges using educational balls, presents a promising strategy for enhancing both cognitive and motor creativity in children. This study aimed to assess the effectiveness of an Eduball-based PE program in supporting creativity and motor fitness in early school-aged children.

**Methods:**

The study involved 173 primary school children (48% girls) aged 8–9 years. Cognitive creativity was assessed using the Test for Creative Thinking–Drawing Production (TCT-DP). Motor creativity (fluency, originality, imagination) was evaluated using the “Thinking Creatively in Action and Movement” (TCAM) test. Motor fitness was assessed using selected Eurofit battery tests: a 20-meter Shuttle Run to evaluate cardiorespiratory endurance, and a 10 × 5-meter Shuttle Run (SHR) to assess speed and agility. The Piórkowski apparatus (AP) test measured hand-eye coordination, reaction time, and precision of movements. The eight-week intervention used the Eduball method during PE classes in two experimental groups: Experimental Group 1 (EG1) had one Eduball session per week; Experimental Group 2 (EG2) had two. The control group (CG) participated only in traditional PE classes. Pre- and post-tests were analyzed using one-way ANOVA on ranks and the Wilcoxon signed-rank test.

**Results:**

At the pre-test there were no significant differences between groups in any measured parameter. However, statistically significant differences were observed in the post-test for the TCT-DP scores, motor imagination (TCAM), and eye-hand coordination in AP test, all favoring EG2. Within-group comparisons showed significant improvements in all motor fitness parameters, as well as in TCAM fluency and imagination across all groups. However, no significant change in TCT-DP or TCAM originality was observed in the experimental groups. The control group showed a significant decline in these two parameters.

**Conclusion:**

The Eduball method significantly supported selected aspects of creativity and motor fitness among 8–9-year-old children. These findings highlight the method’s potential as an effective pedagogical tool for fostering creativity development through physical education in school settings.

## Introduction

In rapidly evolving world, where technological advancements, constant change, and increasing competition across all areas of social life fuel the pace of leaving, creativity has become an essential and highly valued skill. It is now considered a crucial asset for an employee at today’s labor market. According to the latest Future of Jobs Report 2025 from the World Economic Forum (1), creativity is among the top five most in-demand professional skills in the workplace. This highlights the importance of having an agile, innovative, and collaborative workforce, where creative abilities play a significant role in achieving success.

Prior research has shown, that creativity has been viewed from a multidimensional perspective, recognizing its expression not only in art but also in science, engineering, commerce, and business innovation (2–4). In this regard, it becomes quite understandable the fact that creativity, while long recognized as an important focus in education, has in recent years gained renewed and increasing attention (5, 6) as a key factor in preparing the younger generation for adult life and its growing competitive demands.

In recent years more and more research has been devoted to investigating methods and strategies for developing children’s creativity in educational environment (6–10). Despite this, few studies in this context focus on PE classes and their potential (11–14). Positive impact on children’s creativity should become a core of their schooling and sound development.

Most methods designed to develop children’s creativity look toward the chances offered by more academic subjects (6, 15), while PE remains underemphasized in this regard. This is due to the prevalent stereotype that PE is solely intended for the development of motor skills and improvement of children’s physical fitness. This misconception arises from the fact that modern PE classes are primarily based on their reproductive nature from children’s side, emphasizing motor skill acquisition while predominantly focusing on physical fitness and the repetition of existing motor patterns (16–18). It is important to recognize that early school-aged children are in period of rapid motor development, during which both fundamental motor skills (e.g., running, jumping, throwing) and underlying motor abilities (such as strength, speed, agility, coordination and balance) become more refined (19–21). At this stage, children show marked improvements in movement efficiency, allowing them to combine and apply skills in increasingly complex ways, particularly in play contexts. Aerobic capacity and speed demonstrate particularly strong developmental gains in this age range, reflecting both biological maturation and increased engagement in structured activity (22). In addition, fine motor skills progress steadily, leading to greater dexterity and enhanced eye-hand coordination. These developmental changes support better overall coordination and balance, which are critical for successful participation in organized games and physical activity (20, 21). This natural tendency toward rapid motor development should be stimulated accordingly via the design of PE programs.

However, the potential of PE extends far beyond these aspects. This assertion is supported by empirical evidence dating back over a decade, particularly in Europe. Heilmann and Korte’s (23) content analysis of European school curricula identified PE among the top three subjects with the highest frequency of creativity-related terms, underscoring its long-recognized potential for fostering creativity in school settings. PE offers activities that naturally allow creative potential to emerge, including team games, where students express creativity by modifying game rules, discovering alternative movements, and demonstrating divergent approaches to sports skills. Such characteristics make PE particularly well-suited to develop creative capacity through movement (24).

Movement is a necessity and natural stimulus for children from birth (25), while play is one of the most engaging and fundamental activities in their development (26, 27). Research shows that creativity is less likely to flourish in a state of boredom or negative emotions. On the contrary, it tends to grow in environments characterized by positive mood and engagement (28, 29). PE teachers, leveraging the unique nature of their subject, can foster such an atmosphere by creating a positive, engaging learning environment. Within such a setting, creativity may be effectively supported and expressed through movement, making its development feel enjoyable and effortless rather than burdensome.

The traditional way of defining creativity is as the ability to produce something both new (original) and appropriate to the task or domain (30). Another widely used definition describes creativity as the capacity to generate ideas or products that are both novel and useful (31). However other perspectives frame creativity not as an individual trait or product, but as a systematic, embodied, and socioculturally situated phenomenon emerging from dynamic interactions between individuals and their environments (32). In this study, we examine creativity within the school context, specifically little-c creativity—the everyday creative potential that can be cultivated through educational experiences and that differs from the eminent, domain-transforming creativity associated with professional expertise or genius (33). Within the 4C framework (33), little-c creativity lies between mini-c (personally novel insights) and pro-c (professional-level achievements). It includes everyday behaviors such as pretend play, problem-solving, or questioning, and is vital for children’s learning, wellbeing, and development (34).

Creativity in movement, or motor creativity, has been cautiously defined by Wyrick (35) as the ability to produce both varied and unique motor responses to a stimulus (35), with the important caveat that its objective assessment presents considerable difficulties. While Wyrick emphasized variety and uniqueness, later approaches, such as Torrance’s Thinking Creativity in Action and Movement (TCAM) (36)—expended this conceptualization by also incorporating fluency (the number of responses) as e separate criterion. Accordingly, while creativity can be conceptualized in terms of products, processes, or broader systemic perspectives (37), our study adopts a primarily product-oriented approach, focusing on observable children’s outcomes of both cognitive and motor creativity.

Understanding motor creativity is particularly relevant in school-based PE. Studies examining the effectiveness of methods for developing motor creativity in PE classes have confirmed their positive impact (11, 12, 38). However, researchers emphasize that traditional PE classes that primarily focus on physical fitness and the repetition of existing motor skills do not effectively promote motor creativity. Traditional PE classes are typically based on reproductive teaching styles, in which teachers direct activities, emphasize repetition, and prioritize mastery of established motor patterns rather than fostering creative exploration (17). In practice, such classes often rely on linear, teacher-centered instruction with standardized exercises, where the main goal is physical fitness and skill automatization rather than problem-solving or innovation. Instead, motor creativity mainly emerges from activities that encourage nonlinear mechanisms of learning. Strategies such as constraint manipulation (e.g., performing a movement using only one limb), functional variability (e.g., “show me another way to do this”), problem-solving (e.g., “find a way to…”), improvisation (e.g., “do whatever you want”), fantasy play (e.g., “pretend you are an animal”), and creation (e.g., “invent a completely new movement”) offer significantly greater potential for developing motor creativity (11). In turn, in linear approaches, task constraints are often too rigid, limiting children’s ability to explore new movement possibilities (11, 39, 40). The lack of effectiveness in stimulating motor creativity within the traditional PE curriculum points to a broader challenge in the PE system, where the prevailing focus on standardized performance often suppresses opportunities for innovation and creative impression. Based on this, it can be assumed that fostering motor creativity in PE classes requires a specifically designed PE method or strategy.

One of the promising and innovative methods in PE is the Eduball method—a unique educational approach that integrates physical activity (PA) with academic learning. The method integrates PA with subject-matter learning through structured lesson plans, interdisciplinary instructional strategies, and defined didactic principles. This method enhances children’s cognitive abilities by combining movement and play, making the learning process more engaging and effective (41). It was developed in 2001 by Polish academic researchers Rokita and Rzepa. The concept is based on an interdisciplinary approach to teaching PE, blending cognitive engagement with movement tasks. This is achieved through the use of special didactic tools—educational balls (Eduballs)—which are incorporated into PE classes with modified tasks that stimulate cognitive skills and functions during movement (42).

The Eduball set consists of 100 balls in five colors (red, blue, green, yellow and orange), each marked with letters, numbers or mathematical symbols (43, 44). Through interactive physical activities with Eduballs, children learn about colors, letters, numbers and fundamental mathematical operations, as well as various language and mathematical rules. At the same time, they develop fine and gross motor skills, along with fundamental movement abilities such as passing, catching, dribbling, throwing, rebounding, and receiving the ball (41). Plays and games with Eduballs are based on natural forms of movement and holistically stimulate children’s development. The numbers, letters, and symbols, along with colors of the educational balls, allow for their broad application in teaching and reinforcing concepts from nearly all school subjects during PE classes (42, 45, 46). Since the introduction of educational balls, numerous pedagogical studies have been conducted to assess the effects of movement-based learning with Eduballs (47–53). Research has demonstrated that PA involving educational balls have a positive impact on overall body coordination, eye-hand coordination, spatial–temporal orientation, and locomotor skills of primary school students (48, 50).

However, the most distinctive feature of the Eduball method is its influence on pupil’s academic performance. Studies have confirmed a significant impact of the method on children’s language skills (both native and foreign), reading and writing abilities, and mathematical competencies (47, 49, 52, 53). Additionally, positive trends have been observed in the graphomotor efficiency of primary school students (51). The link between cognitive abilities and academic performance has been well established (54, 55), with evidence indicating that this relationship is particularly strong at the primary school level compared to later stages of education (54). Moreover, Runco (56) demonstrated that basic cognitive process—including attention, perception, memory, information processing—are directly associated with creative problem solving.

Based on the concept of the Eduball method, it can be therefore assumed that it offers a valuable opportunity to foster children’s creativity within PE setting. Importantly, this potential may arise not only indirectly through its well-documented influence on academic performance and, consequently, on cognitive function—which are closely linked to creativity—but also directly through the nature of Eduball tasks themselves. These tasks are purposefully designed to create simultaneous cognitive and movement challenges, encourage nonlinear movement patterns and engage pupils in problem-oriented activities. This assumption, supported by previous research findings (47, 49, 51, 53, 57) led to the hypothesis that the Eduball method could be an effective approach for fostering both children’s cognitive and motor creativity in PE classes.

To test this a study was designed to evaluate the impact of the Eduball method on the development of cognitive creativity and motor creativity among early-age school pupils in PE settings. To achieve this goal, a specially designed eight-week intervention program using the Eduball method was implemented in PE classes for second-grade primary school pupils. The hypotheses of this study were as follows: (1) a specially designed educational program incorporating the Eduball method in PE classes will lead to greater improvements in children’s cognitive creativity and motor creativity compared to those participating in traditional PE classes; (2) the impact of PE classes utilizing the Eduball method on children’s motor fitness will be comparable to that of traditional PE classes.

## Materials and methods

### Participants

The study recruited 173 healthy children (83 girls and 90 boys) aged 8 to 9 from the second grade of three public primary schools in Poznan, Poland (urban area). The school’s PE curriculum followed the unified standards established by the Polish National Ministry of Education. The sample size calculation was conducted using G*Power 3.1 software, with an anticipated effect size of 0.25, a significance level (α) of 0.05 and statistical power set at 0.90, which resulted in a minimum sample size of 162. The research was carried out between January and May 2024. Exclusion criteria for participants were: (1) children younger than 8 and older than 9 years; (2) children with congenital diseases; (3) children with intellectual developmental disorders or learning disabilities. The descriptive characteristics of the sample groups are presented in Table 1.

**Table 1:** Descriptive characteristics.

Variables		EG1 (*n* = 61)	EG2 (*n* = 39)	CG (*n* = 73)
		*M* ± SD	*M* ± SD	*M* ± SD
Sex	Boys	31 (50.8)	19 (48.7)	40 (54.8)
	Girls	30 (49.2)	20 (51.3)	33 (45.2)
Age (years)		8.05 ± 0.28	8.03 ± 0.16	8.03 ± 0.29
Body height (cm)		132.25 ± 6.93	134.03 ± 6.18	132.56 ± 6.29
Body weight (kg)		30.72 ± 7.99	31.05 ± 5.91	29.02 ± 5.39

M, mean; SD, standard deviation; cm, centimeters; kg, kilograms; EG1, Experimental Group 1; EG2, Experimental Group 2; CG, Control Group.

In addition, information on children’s extracurricular activities was collected, including additional physical activity, organized or competitive sports (e.g., ball games), and non-sport activities such as creative arts. These activities were considered as potential variables influencing creativity. However, the activities were highly varied in type and intensity, and so significant correlations were found between these variables and either cognitive or motor creativity.

The study was conducted in accordance with the Declaration of Helsinki and the study protocol was approved by the Local Bioethics Committee of the Karol Marcinkowski University of Medical Science in Poznan (decision number 400/23 of 11 May 2023). Written informed consent was obtained from the parents or guardians of the child participants and verbal assent was also obtained from the children prior to participation.

### Study procedure

Pre- and post-assessments were conducted in two experimental and one control groups. The assessments included measurements of anthropometric data (body weight and height), creativity, motor creativity, and motor fitness level. All measurements and tests were performed in a school setting (gym and classrooms). First, the creativity test was conducted in a classroom. Then, motor fitness and motor creativity tests were conducted in a school gym. The participants were informed about the test procedure and each test item was accompanied by detailed instructions. Creativity assessments were conducted by trained instructors under the supervision of a qualified psychologist, and motor fitness assessments were conducted by trained research specialists to ensure reliable measurements.

### Assessment of anthropometric parameters

The measurement of body weight and height was conducted using anthropometric instruments (Wunder Sa. Bi. Srl., Milan, Italy) in accordance with the prevailing standard methodology. Participants were barefoot during the measurement. Weight was measured to the nearest 0.1 kg, while the participants wore a minimum of clothing. Height was measured to the nearest 0.1 cm. The participants were instructed to adopt an upright posture, maintain a forward-facing gaze, and keep their knees straight, with their arms at their sides. All measurements were taken once by trained research assistants.

### Creativity assessment

The Test for Creative Thinking-Drawing Production (TCT-DP) was used to assess pupils’ creativity following Urban’s protocol (58). The participants were asked to complete a drawing that begins with a square frame and six figural fragments placed on a test sheet following the Jellen and Urban method (59, 60). Additionally, the subjects were asked to provide a title for the drawing. It has been previously reported that eventual experience in drawing is unrelated to TCT-DP score (58). All pupils performed this test on their own. The instructions emphasized freedom of expression, and the participants were not informed about the time limit (although the maximum allowed time was 15 min, which was considered in the scoring) (58–60). The assessment of the TCT-DP consisted of fourteen criteria, which include the following: (1) continuations, (2) completions, (3) new elements, (4) connections made with a line, (5) connections that contribute to a theme, (6) boundary breaking that is fragment-dependent, (7) boundary breaking that is fragment-independent, (8) perspective, (9) humor and affectivity, (10) unconventionality with, manipulation of the test material, (11) unconventionality with, abstract elements, (12) unconventionality in the use of symbols, (13) unconventionality with unconventional usage of the given fragments, and (14) speed (58). A qualified psychologist assessed the creativity scores with the final TCT-DP result calculated as the total sum of points obtained across all the criteria.

The test has two versions (A and B) differing only in the positioning of the elements relative to the test item. In version B, the initial arrangement is rotated 180 degrees. In this study version A was used for the pre-test, while version B was used for the post-test. The reliability and validity of the TCT-DP have been confirmed in numerous studies (58, 60, 61). In Polish standardization studies internal consistency was assessed using Cronbach’s alpha, with coefficient ranging from 0.62 (army cadet school students) to 0.80 (preschool children) (60).

### Motor creativity assessment

Torrance’s “Thinking Creatively in Action and Movement” (TCAM) test was used to assess pupils’ motor creativity (36). The test administration and scoring guide were translated from English to Polish using back-to-back translation method with appropriate adaptations. The test included four activities. In the first activity, the child was asked to cover the designated distance of three meters in as many different ways as possible. The second activity required the participant to imagine themselves in six fiction situations and perform appropriate actions accordingly. For the third activity the participant was asked to demonstrate or describe as many different ways as possible to place a paper cup into wastebasket located two-meter away. In the fourth activity the subject was asked to list or demonstrate different possible uses for a paper cup (36). The first, third and fourth activities were scored in two categories, motor fluency and originality, while second activity assessed imagination. Motor fluency is determined as the ability to create different, alternative movement patterns and was scored by the total number of ways and combinations of movements the child invented. Motor originality is determined by the ability to produce novel, unique, and unusual ways of movement and was scored by comparing the child’s responses with a reference list based on the statistical infrequency of the responses, after which points were awarded accordingly. Imagination is defined by the ability to imagine, empathize, fantasize, and assume unaccustomed roles and was assessed on a five-point rating scale (from 1 = no movement to 5 = excellent imitation) for each fiction situation.

All scoring was conducted in accordance with the original manual (36). Overall testing time ranged between 10- and 30-min per child. The reliability coefficients for the individual activities are 0.71 for the first activity, 0.79 for the second activity, 0.67 for the third activity, and 0.58 for the fourth activity. The overall reliability coefficient for the Torrance TCAM test is 0.84 (36).

### Motor fitness assessment

Participants’ motor fitness level was assessed using selected tests from the Eurofit battery (Council of Europe, Committee for the Development of Sport, Strasbourg, France) (62). These included a 20-meter endurance Shuttle Run to evaluate cardiorespiratory endurance, and a 10 × 5-meter Shuttle Run (SHR) to assess running speed and agility. In addition, the Piorkowski test was used to evaluate eye-hand coordination, reaction speed and movements precision.

In the 20-meter Shuttle Run test, participants were asked to stand behind the starting line facing the second line, which was 20 m away. When a special sound signal was heard, they started running. Participants continued running between the two lines turning when the recorded signal sounded. They needed to run at a pace that ensured they reached the end of a 20-meter section when they heard the signal to change direction. Their task was to maintain the pace set by the sound signal for as long as possible. The test ended when a participant could no longer keep up with the signals or feels too tired to continue. The score was determined as a number of levels completed, based on the number of 20-meter shuttles reached before the participant was unable to keep up with the recorded sound signal. The final score was the last level completed (62).

The 10 × 5 m SHR test involved participants running back and forth over a 5-meter distance, changing direction 10 times, to measure speed and agility. The score was based on the time it took for the participant to complete this task (62).

The Piórkowski test was conducted using the Piórkowski apparatus (AP) (Psychology laboratory “Driver,” Ustrzyki Dolne, Poland). The apparatus has 10 buttons arranged in one row with a LED above each button that lights up to indicate which button to press. Only one LED lights up at a time. The task was for the participant to press each subsequent button indicated by the apparatus using both hands. The right hand was to be used for buttons on the right side, and the left hand for buttons on the left side. The device does not wait for the correct press but sets its own pace, and the participant were required to hit the button correctly as many times as possible. In this study, the parameters included a 60-s period with a stimulus presentation frequency of 30 pulses. The outcome was measured by the number of correct responses. Thorough instructions were always provided before the examination. The test was previously conducted under Polish conditions in studies by Tomczak et al. (63) and Merkisz et al. (64) and proved reliable.

### Intervention program

The pre-test, which was conducted between January and February 2024, utilized the aforementioned testing methods. Following this assessment, entire classes of participants were randomly divided into three groups: two experimental groups (EG1 and EG2) and one control group (CG). Randomization was performed at the class level to ensure that all children within a class received the same intervention. The intervention program commenced in March and was implemented over eight weeks under natural school conditions in two experimental groups. The experimental factor was a specially designed PA program implemented into PE classes. This program, based on plays and games using the Eduball set, aimed to stimulate children’s creativity.

In accordance with the Polish PE curriculum for second grade, pupils are scheduled to have three PE classes per week. In this study, EG1 participated in the Eduball intervention program once per week, alongside two traditional PE classes. EG2 engaged in the Eduball program twice a week, with one traditional PE class. Meanwhile, CG followed the standard curriculum, receiving only traditional three PE classes without Eduball intervention. The purpose of creating two experimental groups was to compare the effect of the intervention program based on the frequency of Eduball sessions.

A total of 8 Eduball-integrated PE classes were conducted for EG1, while EG2 received 16 such classes. Each 45 min class was structured into three parts: an introduction (5 min), a main part (35 min), and a concluding synopsis (5 min). The main goal of each class was to stimulate pupils’ creativity through movement-based problem-solving tasks using the Eduball method. These activities encouraged children to: create new movement patterns, generate multiple effective strategies for completing tasks, solve problem-based scenarios that combined movement and cognitive challenges in a nonlinear way. The mathematical numbers placed on the educational balls allowed for creation of tasks that stimulated creative thinking in solving mathematical operations, while the alphabet letters on the Eduball enabled plays and games that fostered creative thinking in word formation, sentence construction, and storytelling. Additionally, the five-color design of the balls enhanced the creation of tasks that promoted children’s creativity.

The scenarios for PE classes using Eduball were developed based on an established pool of Eduball examples [(46) (PDF available online), (65)]. Furthermore, some sample scenarios were presented in recent open-access Eduball studies (47, 51). These sample scenarios provide a detailed description of common Eduball-based activities, including their objectives and the specific skills they are aimed to develop. In this intervention, some scenarios were used in their original form, while others were modified to alight with specific goals of this study.

Here are examples of some scenarios used in Eduball-intervention PE classes:

Scenario 1. The pupils are divided into four teams. They gather in four corners of the gymnasium. The balls are spread in the center circle of the basketball court. The pupils have to form any creation using of the specific features of the Eduballs (their colors, letters, numbers, mathematical signs, etc.). To move the necessary balls to their corners, the pupils need to cooperate. They may pass the ball from one to another, but they cannot move while carrying them. Only one ball at a time can be transported. They have five minutes to complete the task. At the end of the game, each team presents their work to the other teams, which try to determine what the creations are (65).

Scenario 2: The pupils are divided into two teams (yellow and green team). Each team gets a ball with the number “8” and places it on a rubber ring. Their task is to form as many mathematical operations involving addition, subtraction, multiplication and division as they can and resulting in “8.” During the activity, the pupils are not allowed to carry the balls with their hands. They have 5 min to complete the task. Then, the teacher checks the correctness of the equations (65).

Scenario 3: Each pupil stands in the center circle of the basketball court. The teacher stands next to the balls in five colors on the rubber rings in front of students. When the teacher lifts a certain ball, the pupils have to behave and move according to the weather conditions, e.g., yellow—sun, blue—rain, green—wind, red—storm, orange—sunset. Variation: The pupils are divided into 5 groups. Each team has to announce and present the weather forecast using the balls in all the colors (65).

Scenario 4: The pupils have green or yellow balls. They move to the rhythm of the music and play with their ball. When the music is turned off, they form three-to-four-person groups. They have to create and write a sentence consisting of 3 or 4 words beginning with the letters on the balls, e.g., the pupils have “s,” “b,” “h” and “t,” and they might form the sentence “She has two brothers” or “Ben sold this house” (65).

Scenario 5: The pupils are divided into four teams. The balls (fruits and vegetables) are spread all over the gym. The pupil’s task is to make a fruit or vegetable salad. Each team moves as one body, holding hands, and collects as many balls as they can gather to their base. Then, they count the balls and state their salad ingredients, e.g., “Our fruit salad consists of 7 avocados, 3 strawberries, 1 plum, 1 orange and 12 bananas,” “The ingredients of our vegetable salad are 2 carrots, 2 eggplants, 11 cucumbers, 1 tomato and 8 onions” (65).

After the eight-weeks intervention program, a post-test was conducted for all groups in May. The data of participants of experimental groups who attended less than 60% of intervention program (fewer than 5 of 8 sessions in EG1 or fewer than 10 of 16 sessions in EG2) were excluded and not considered in the analysis of the experimental results. All Eduball sessions were conducted by previously trained PE teachers under the direct supervision of the project coordinator, and each session followed standardized, carefully designed scenarios. This approach ensured consistent delivery of the intervention maintained methodological rigor, and minimized variability, thereby guaranteeing the fidelity and integrity of the intervention throughout the study.

### Statistical analysis

Statistical analyses were performed for creativity and motor fitness variables. After assessing normality using the Shapiro–Wilk test, the lack of normality of distribution was noticed. Therefore, to compare differences in creativity and motor fitness variables between experimental and control groups the one-way ANOVA on ranks (Kruskal-Wallis’s H-test) was employed. The comparison of pre- and post-test results within the experimental and control groups was conducted using the Wilcoxon signed-rank test. The effect size was calculated for each statistical test used and interpretated based on the corresponding scale: Kruskal-Wallis’s test: Small - 0.01, Medium - 0.06; Large - 0.14; Wilcoxon signed-rank test: Small - 0.1, Medium - 0.3, Large - 0.5 (66–68).

For statistical testing, Statistica 13.3. was used (Statsoft, Kraków, Poland), and statistical significance was set at *p* < 0.05.

## Results

A comparison of creativity variables between the experimental and control groups at pre- and post-test is presented in Table 2. At the pre-test no statistically significant differences were found among the three groups in any of the creativity variables (TCT-DP score, TCAM: fluency, originality, imagination). However, statistically significant differences were observed in the post-test for the following parameters: TCT-DP test—significant differences were found between EG1 and EG2, and between CG and EG2, both in favor of EG2 (*p* < 0.01, effect size = 0.09); imagination (TCAM)—a significant difference was observed between EG1 and EG2, favoring EG2 (*p* < 0.01, effect size = 0.05). The within-group comparison of pre- and post-test results (Table 2) showed statistically significant improvement in fluency (TCAM) for all groups (effect size = 0.4). Imagination (TCAM) also improved significantly in all groups, with the largest improvement observed in EG2 (effect size = 0.7), while gains for EG1 and CG were comparable (effect size = 0.5). No significant changes were observed in TCT-DP and originality (TCAM) within EG1 or EG2; however, the effect size for TCT-DP in EG2 was 0.3, indicating a moderate practical improvement despite the lack of statistical significance. In contrast, CG showed statistically significant negative changes in TCT-DP (effect size = 0.2) and originality (effect size = 0.3).

**Table 2:** Comparison of median test scores for creativity variables between experimental and control groups (*n* = 173) at pre- and post-test.

Variables		EG1 (*n* = 61) 1	EG2 (*n* = 39) 2	CG (*n* = 73) 3	Between-group *p* values			η^2^
					1 vs.2	1 vs.3	2 vs.3	
TCT-DP (pts)	Pre-test Mdn (Q1–Q3)	21 (16–30)	27 (16–34)	22 (16–29)	ns	ns	ns	0
	Post-test Mdn (Q1–Q3)	21 (16–30)	30 (21–27)	19 (15–25)	<0.01	ns	<0.01	0.09
	*p*	ns	ns	<0.05	–			
	*r*	0	0.3	0.2				
TCAM, Fluency (pts)	Pre-test Mdn (Q1–Q3)	22 (18–31)	22 (15–30)	21 (16–27)	ns	ns	ns	0
	Post-test Mdn (Q1–Q3)	27 (22–37)	25 (21–47)	25 (18–35)	ns	ns	ns	0
	*p*	<0.05	<0.05	<0.05	–			
	*r*	0.4	0.4	0.4				
TCAM, Originality (pts)	Pre-test Mdn (Q1–Q3)	22 (17–35)	25 (14–35)	24 (14–35)	ns	ns	ns	0
	Post-test Mdn (Q1–Q3)	25 (15–33)	23 (16–35)	18 (14–30)	ns	ns	ns	0
	*p*	ns	ns	<0.05	–			
	*r*	0.1	0.1	0.3				
TCAM, Imagination (pts)	Pre-test Mdn (Q1–Q3)	18 (15–21)	19 (15–22)	19 (15–22)	ns	ns	ns	0
	Post-test Mdn (Q1–Q3)	21 (19–24)	26 (21–30)	22 (19–27)	<0.01	ns	ns	0.05
	*p*	<0.05	<0.05	<0.05	–			
	*r*	0.5	0.7	0.5				

*p* < 0.05; Mdn, median; pts, points; TCT-DP, Test for Creative Thinking-Drawing Production; TCAM, Torrance’s “Thinking Creatively in Action and Movement” test; EG1, Experimental Group 1; EG2, Experimental Group 2; CG, Control Group. η^2^ (effect size - Wilcoxon test): Small - 0.01, Medium - 0.06, Large - 0.14 and above.

*r* (effect size - ANOVA): Small - 0.1, Medium - 0.3, Large - 0.5 and above.

A comparison of motor fitness variables between the experimental and control groups at pre- and post-test is presented in Table 3. At the pre-test no statistically significant differences were observed among the three groups in any motor fitness variable (20 m Shuttle Run, 10 × 5 m SHR, AP test). Similarly, post-test comparison showed no significant differences between groups in 20 m Shuttle Run and 10 × 5 m SHR tests. However, post-test comparison revealed a statistically significant difference in the AP test between EG1 and EG2, and between CG and EG2, both in favor of EG2 (*p* < 0.001, effect size = 0.09). Within-group comparison (Table 3) indicated statistically significant differences in all motor fitness parameters across all the three groups indicating an improvement in post-test. The largest improvement was observed in the AP test for EG2 (effect size = 0.7), while gains for EG1 and CG were comparable (effect size = 0.4).

**Table 3:** Comparison of median test scores for motor fitness variables between experimental and control groups (n = 173) at pre- and post-test.

Variables		EG1 (*n* = 61) 1	EG2 (*n* = 39) 2	CG (*n* = 73) 3	Between-group *p* values			η^2^
					1 vs.2	1 vs.3	2 vs.3	
20 m Shuttle Run (lvl)	Pre-test Mdn (Q1–Q3)	3 (2.4–4.1)	2.5 (2.2–4.1)	3 (2.2–3.6)	ns	ns	ns	0
	Post-test Mdn (Q1–Q3)	3.4 (2.4–4.5)	3.5 (2.7–4.2)	3.3 (2.4–4.7)	ns	ns	ns	0
	*p*	<0.05	<0.05	<0.05	–			
	*r*	0.3	0.6	0.6				
10 × 5 m SHR (s)	Pre-test Mdn (Q1–Q3)	24.4 (23.2–26.6)	25.1 (23.9–26.2)	25.2 (23.3–27.1)	ns	ns	ns	0
	Post-test Mdn (Q1–Q3)	23.1 (21.9–25.0)	23.7 (22.3–26.1)	24.2 (21.8–25.3)	ns	ns	ns	0
	*p*	<0.05	<0.05	<0.05	–			
	*r*	0.4	0.6	0.5				
PA (no/30 pulses)	Pre-test Mdn (Q1–Q3)	25 (21–27)	26 (23–28)	25 (22–27)	ns	ns	ns	0
	Post-test Mdn (Q1–Q3)	26 (24–28)	28 (27–30)	27 (25–28)	<0.01	ns	<0.01	0.09
	*p*	<0.05	<0.05	<0.05	–			
	*r*	0.4	0.6	0.4				

*p* < 0.05; Mdn, median; lvl, level; s, seconds; no, number; SHR, 10 × 5 m shuttle run; PA, Piorkowski apparatus; EG1, Experimental Group 1; EG2, Experimental Group 2; CG, Control Group.

η^2^ (effect size - Wilcoxon test): Small - 0.01, Medium - 0.06, Large - 0.14 and above.

*r* (effect size - ANOVA): Small - 0.1, Medium - 0.3, Large - 0.5 and above.

## Discussion

The aim of this study was to examine the impact of an eight-week educational intervention program based on the Eduball method in PE classes on the development of cognitive creativity, motor creativity, and motor fitness in second-grade pupils. The findings indicated that the Eduball intervention had a positive influence, particularly on creativity-related outcomes, with some variability depending on the specific variable assessed and the frequency of the stimuli.

In terms of cognitive creativity, as measured by the TCT-DP test, the implementation of the Eduball-based activities in PE classes demonstrated a positive impact on this parameter in both experimental groups. Specifically, the findings revealed the emergence of statistically significant difference in post-test creativity score between groups, favoring EG2 (the one with more frequent stimuli). Although the within-group improvement in EG2 was not statistically significant, the effect size indicated a moderate effect, suggesting a potentially meaningful change that could reach statistical significance with a larger sample size. EG1 demonstrated a stable creativity score levels over time, while a statistically significant decrease in creativity was observed in CG. A possible explanation for the observed decline in creativity in the CG may be related to the lack of specific conditions that stimulate and support creativity. Previous research has shown that during the transition from early to middle childhood (ages 8–10), children increasingly shift from a spontaneous, preconventional mode of thinking toward more conventional and socially orientated responses, which may limit the expression of creativity in structured school contexts (69–71). In traditional educational settings, academic success is often associated with accuracy and rule compliance, while original responses are less encouraged (72). This tendency may suppress children’s willingness to take risks and generate novel ideas, even if their creative potential remains intact. Moreover, as school demands increase, children may prioritize strategies that emphasize correct answer over exploratory thinking, which can reduce observable creativity scores (73). Thus, the decline in creativity observed in CG may reflect the combined influence of developmental trends and the convergent orientation of traditional schooling. At the same time, the present results can also be interpreted in line with the “if you do not use it, you lose it” principle: children who did not engage in systematic creativity-promoting activities (as in the Eduball intervention) showed a measurable decline in creativity over time. This interpretation is consistent with previous findings emphasizing the importance of regular stimulation and practice in maintaining and enhancing creative abilities (56, 74). The observed pattern—decline in CG, stability in EG1, and improvement in EG2—suggests that exposure to enhanced creativity-oriented challenges is definitely required if the cultivation of the creative potential of children under development is a desirable educational objective. The dose–response relationship suggests that, to prevent loss of creative potential during this critical period, children must receive at leas minimum dosage required for stability, while enhanced exposure produces meaningful improvements. In line with previous studies by Richard et al. (11), Bournelli and Mountakis (75) these fundings demonstrate that creativity requires active cultivation during this sensitive developmental period to prevent natural decline, and creativity-oriented movement activities like the Eduball method represent an effective means of achieving this objective. Taken together, these findings underscore the importance of systematically cultivating creativity, while highlighting the added value of higher intervention frequency, as evidenced by the favorable outcomes in EG2.

Regarding motor creativity, the TCAM test revealed varied effects across different motor creativity variables. In this study, pupils from CG, who participated in traditional PE classes, as well as those from EG1 and EG2, who took part in an eight-week Eduball intervention program, demonstrated similar improvements in motor fluency. This finding suggests that motor fluency at this age has a natural tendency to develop, as previously reported by Domínguez et al. (76). The Eduball intervention did not produce an additional effect within the eight-week period. In contrast, Richard et al. (11), in a study examining the impact of a nonlinear pedagogy-based PE program on the motor creativity of nine-year-old children over a three-month intervention period (10 sessions), reported that while there were no significant differences in motor fluency between the groups in the pre-test, a statistically significant difference emerged in the post-test, favoring the experimental group. Similarly, a study by Ourda et al. (77), investigating the effect of motor creativity intervention conducted over one academic semester with 4- to 5-year-old children (20 sessions), showed a significant increase in motor fluency in the experimental group while no statistically significant change was observed in the control group. These discrepancies may be explained by differences in the nature of the intervention programs, as the cited studies may have included activities more directly targeting motor fluency, whereas the Eduball program was designed to stimulate creativity through a broader set of cognitive-motor challenges. Additionally, differences in intervention duration may also account for these divergent results. It therefore seems that enhancing motor fluency through the Eduball intervention may require a period longer than eight weeks to achieve significant improvements. Moreover, fluency, although frequently included in creativity assessments, has been described as the most challenging and the least strongly associated with creativity (compared to, for example, originality) and is not recommended as a standalone indicator (78). These considerations help explain the absence of additional intervention effects on fluency despite improvements across all groups.

In contrast, motor originality variable in this study demonstrated a statistically significant decrease in CG, while the levels in both experimental groups remained unchanged. However, unlike the present study, the research of Asadi et al. (79), which examined the effect of nonlinear pedagogy on the motor creativity of 7-year-old children over a six-week intervention conducted three times per week, reported no significant changes in the control group, but a significant increase in the experimental group. Research by Domínguez et al. (76) indicated that motor originality naturally increases between the ages of 6 and 8, but tends to decline between the ages of 8 and 10. In this context, the observed decrease in motor originality in the CG in the current study with its absence in Asadi et al. (79), may be attributed to these age-related developmental changes. Thus, the implementation of nonlinear pedagogy in Asadi’s study contributed to an additional enhancement of motor originality, complementing its natural developmental trend at this age. In contrast, the Eduball method in our study acted as a protective factor, preventing the age-related decline in motor originality and helping to maintain its level in both experimental groups. Notably, in this case, the frequency of Eduball classes per week did not appear to significantly impact the outcomes, suggesting that even a lower frequency of Eduball PE classes may be effective in preserving motor originality during this critical developmental stage in early-age school children. The similar stability of the results in EG1 and EG2 suggests that even one Eduball session per week may be sufficient to prevent a decline in this creativity parameter. However, it may not necessarily lead to an increase in motor originality over a short period, and a longer implementation period may be required to achieve more pronounced effects. Additionally, the differences across studies highlight certain discrepancies in findings, which may be explained not only developmental factors, but also by differences in assessment tools and scoring procedure. For example, variations between Bertsch’s test (80), used in Asadi et al. (79), and the TCAM, used in the present study, involve differences in tasks, originality scoring criteria, and potentially outdated scoring norms.

Motor imagination, another variable of motor creativity, demonstrated statistically significant improvements across all three groups. However, the effect size for EG1 and CG was identical, while for EG2 it was notably higher. This finding aligns with the research by Domínguez et al. (76), who identified a natural tendency for the development of motor imagination between the ages of 8 and 10 years. This developmental trend was observed across all three groups in the present study. However, the additional improvement observed in EG2 suggests that the Eduball method may have had a positive impact on enhancing motor imagination in this group. Similarly, a study by Alper and Ulutaş (81), which examined the impact of a 12-week creative movement program (conducted twice weekly) on the creativity of 5-6-year-old children, revealed a statistically significant difference in post-test motor imagination scores between the experimental and control group, favoring the experimental group. Comparable results were reported in a study by Ourda et al. (77), which demonstrated a statistically significant difference in motor imagination between the experimental and control groups among 4- to 5-year-old children after 20 sessions of a motor creativity intervention program.

Previous studies investigating the effectiveness of various methods for developing motor creativity allow only limited comparison of changes in motor imagination for the two main reasons. First, some studies (11, 38, 39) employed different tests for motor creativity assessment that did not specifically measure motor imagination. Second, although other researchers (12, 77, 81, 82) employed the same test (TCAM), they focused on preschool-aged children (ages 3 to 6), which is critical factor and does not allow for a direct comparison. As a result, the specific impact of the Eduball method on motor imagination in older children cannot be directly contrasted against the other methods. From this perspective, the findings of the present study regarding motor imagination in 8- to 9-year-olds may be considered both novel and valuable for future research. At the same time, the criterion of imagination in TCAM should be interpreted with caution, as its relationship to creativity is debated and findings remain inconsistent (83, 84). Unlike fluency and originality, imagination is assessed only in TCAM among the main tests of motor creativity, which highlights both its potential value and its conceptual limitations. Therefore, the results on motor imagination should be regarded as complementary to other indicators of creative potential rather than as standalone evidence. Nevertheless, our findings confirm the positive impact of Eduball-based PE classes on children’s motor imagination. Furthermore, the significantly higher effect size in EG2, along with the statistically significant difference in post-test results between EG1 and EG2 in favor of EG2, emphasizes the importance of intervention frequency, suggesting that more frequent sessions resulted in a more positive effect.

Regarding physical fitness, results from 20 m Shuttle Run and 10 × 5-m Shuttle Run tests showed no statistically significant differences in post-test results among the three groups. However, all groups demonstrated significant improvements in these parameters, indicating that both the Eduball method and traditional PE program had a similar effect on the development of children’s physical fitness. These fundings are consistent with studies of Rokita et al. (42, 85), Cichy et al. (86, 87), Pham et al. (88), which showed that PE classes using of Eduball enhance pupils` motor fitness to a comparable extend as traditional PE classes. As for the results of the Piorkowski test, the study revealed a statistically significant difference in post-test in favor of EG2. This finding is in line with previous research of Cichy et al. (48), which demonstrated a more positive impact of Eduball activities on eye-hand coordination compared to traditional PE classes. Furthermore, the statistically significant difference in post-test outcomes between EG1 and EG2, favoring EG2 yet again emphasizes the impact of intervention program frequency, indicating that more frequent sessions had a more pronounced positive effect.

There are several strengths and limitations of this study that should be acknowledged when interpreting its outcomes. First, the sample size of EG2 may have limited the statistical power to detect significant effect—particularly in cognitive creativity, where moderate effect was observed but did not reach statistical significance. Second, the intervention lasted only eight weeks. Previous research suggest that longer intervention may be required to observe significant improvements in motor fluency and originality. Third, participants’ absences from some intervention sessions could have influenced the outcomes, despite applying a 60% attendance threshold for inclusion in the analysis. Fourth, the findings are specific to children aged 8–9 years, which limits the generalizability of the results, especially during this critical stage of creativity development. Fifth, the assessment of creativity was conducted by a single qualified psychologist, with the final TCT-DP result calculated as the sum of points obtained across all criteria. While appropriate blinding procedures were implemented, the single-rater approach may affect assessment reliability. Sixth, although the TCAM test is one of the most widely used measures of motor creativity in children, it presents several notable limitations. In particular, some of its criteria—such as fluency and imagination—have been criticized for their conceptual ambiguity and for their weaker or inconsistent associations with creativity (78, 83, 84). Moreover, TCAM is the only one of the most commonly used motor creativity tests that includes imagination, which makes it both unique and more difficult to compare across studies. Recent literature has highlighted these issues (32) and has called for the development of alternative or complementary approaches to assessing motor creativity (89). Thus, while TCAM remains a popular and practical tool, its limitations should be considered when interpreting the present findings. Finally, the study did not include a follow-up assessment to evaluate the sustainability of the observed improvements over time.

## Conclusion

This study showed that the implementation of the Eduball method in PE classes in second grade pupils had a positive, dose-dependent impact on creativity parameters. Specifically, the enhanced Eduball program (EG2) contributed to improvements in cognitive creativity and preserved motor originality, while the basic program (EG1) helped maintain stability in these parameters. In contrast, the control group (CG) without intervention showed decline in both cognitive creativity and motor originality. Despite the fact that Eduball groups did not demonstrate significant improvements in motor originality, though they avoided the decline observed in the CG. Motor imagination increased in all groups, with EG1 and CG showing comparable gains, while improvements observed in EG2 were higher. Although the Eduball intervention program did not produce additional benefits in motor fluency compared to traditional PE (all groups improved equally), it prevented the deterioration seen in other creativity parameters, suggesting that active creativity-oriented interventions are necessary during this sensitive developmental period. Regarding physical fitness, overall gains were comparable between Eduball and traditional PE; however, eye-hand coordination improved more in EG2, underscoring the added value of higher intervention frequency. Taken together, these findings highlight the potential of the Eduball method as a pedagogical instrument for fostering children’s creative potential in school settings.

## Data Availability

The raw data supporting the conclusions of this article will be made available by the authors, without undue reservation.
